# Emerging Strategies to Enhance Homing and Engraftment of Hematopoietic Stem Cells

**DOI:** 10.1007/s12015-015-9625-5

**Published:** 2015-09-24

**Authors:** Mariusz Z. Ratajczak, Malwina Suszynska

**Affiliations:** Stem Cell Institute at the James Graham Brown Cancer Center, University of Louisville, 500 S. Floyd Street, Rm. 107, Louisville, KY 40202 USA; Department of Regenerative Medicine, Medical University of Warsaw, Warsaw, Poland

**Keywords:** Stem cell homing, Adult stem cells, CXCR4, VLA-4, SDF-1, S1P, C1P, Extracellular nucleotides, Lipid rafts, Priming, Chemotaxis

## Abstract

Successful clinical outcomes from transplantation of hematopoietic stem cells (HSCs) depend upon efficient HSC homing to bone marrow (BM), subsequent engraftment, and, finally, BM repopulation. Homing of intravenously administered HSCs from peripheral blood (PB) through the circulation to the BM stem cell niches, which is the first critical step that precedes their engraftment, is enforced by chemotactic factors released in the BM microenvironment that chemoattract HSCs. These chemotactic factors include α-chemokine stromal-derived factor 1 (SDF-1), the bioactive phosphosphingolipids sphingosine-1-phosphate (S1P) and ceramid-1-phosphate (C1P), and the extracellular nucleotides ATP and UTP. Stem cells may also respond to a Ca^2+^ or H^+^ gradient by employing calcium- or proton-sensing receptors, respectively. In this review, we will present emerging strategies based on ex vivo manipulation of graft HSCs that are aimed at enhancing the responsiveness of HSCs to BM-secreted chemoattractants and/or promoting HSC adhesion and seeding efficiency in the BM microenvironment.

## Introduction

After intravenous infusion, hematopoietic stem/progenitor cells (HSPCs) home through the circulation from peripheral blood (PB) to the bone marrow (BM) stem cell niches in response to chemoattractants secreted in the BM microenvironment, and this process precedes their subsequent engraftment and repopulation of the recipient’s hematopoietic organs [1–3]. It is well known that hematopoietic recovery after transplantation of HSPCs and the final clinical outcome depend on the number and quality of HSPCs present in a graft, which can be estimated in humans by calculating the number of mononuclear cells that express the CD34 antigen. Based on this method, it has been determined that, for transplantation of umbilical cord blood (UCB) with ≤2 human leucocyte antigen (HLA) disparities, the patient has to be infused with ≥2 × 10^5^ UCB-derived CD34^+^ cells/kg body weight [4]. When adult sources of HSPCs are employed (e.g., mobilized autologous PB), 2.5 × 10^6^ CD34^+^ cells/kg body weight is considered a sufficient dose for successful stem cell autotransplant; however, a dose of 5.0 × 10^6^ CD34^+^ cells/kg is considered preferable for achieving early engraftment [5].

These numbers point to the fact that hematopoietic reconstitution and recovery of normal PB counts after hematopoietic transplantation depends on the number of infused HSPCs. On the other hand, it is well known that not all HSPCs infused into the circulation find their way to the stem cell niches in BM, and the majority is trapped in different non-hematopoietic locations in various organs. Therefore, it is important to develop more efficient strategies that improve the seeding efficiency of HSPCs by transplanting them directly to the BM microenvironment [6, 7]. This is a very important issue, in particular when the number of HSPCs in the graft is low, as seen, for example, in adult recipients of UCB when there are low numbers of CD34^+^ cells harvested from BM, or as a result of poor HSPC donor mobilization [6–8]. In all these cases, it is crucial to promote proper homing of HSPCs and thus ensure that as many CD34^+^ cells as possible home to the BM and subsequently permanently engraft.

One of the major mechanisms that retains HSPCs in their BM niches and directs their migration and homing from PB to BM involves interaction of the CXCR4 receptor with α-chemokine stromal-derived factor 1 (SDF-1). While CXCR4 is expressed on the surface of HSCs, SDF-1 is expressed on the surface of cells lining the stem cell niches [1–3, 9]. Homing is also orchestrated by gradients of other chemotactic factors that show chemotactic activity against HSPCs. The list of these chemoattractants is rather short, and so far it has been demonstrated that, besides SDF-1, HSPCs respond to gradients of sphingosine-1-phosphate (S1P) [10–14], ceramide-1-phosphate (C1P) [12], certain extracellular nucleotides, such as ATP or UTP [15], as well as certain ions, such as Ca^2+^ and H^+^ [16, 17].

In this review we present emerging strategies aimed at improving the responsiveness of HSPCs to homing gradients as well as strategies to increase the tethering of transplanted HSPCs to the BM endothelium and subsequently their adhesion in the BM microenvironment. In order to focus on this particular topic, we will not discuss other strategies, such as ex vivo expansion of HSPCs to be used in a graft or application of allo-engraftment-facilitating cells. These strategies that also lead to better engraftment of transplanted HSPCs were recently reviewed elsewhere in excellent publications [18, 19].

**Fig. 1:**
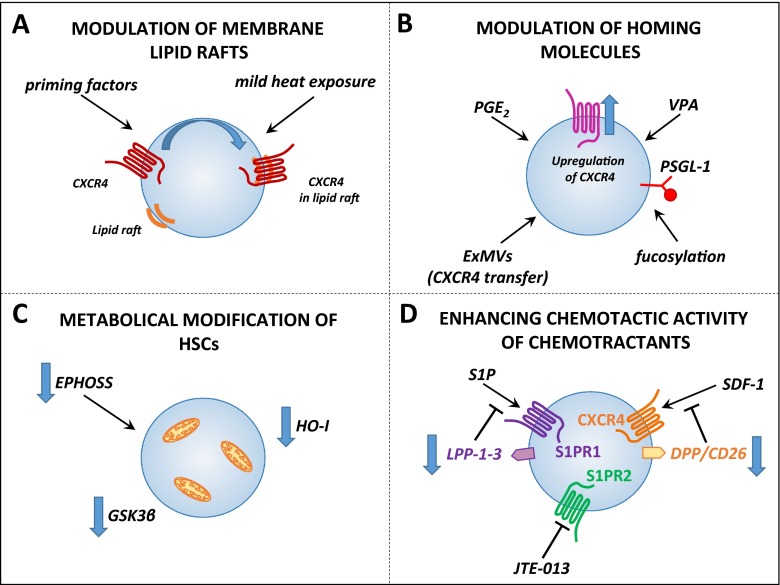
Currently proposed strategies that may improve homing and engraftment of HSPCs. **a** Strategies based on increasing the biological effects of membrane lipid rafts. **b** Modification of the expression and function of molecules involved in adhesion and BM homing. **c** Metabolic modification of the homing properties of HSPCs. **d** Enhancing the bioavailability of chemotactic factors for HSPCs. This classification is presented for reasons of simplicity; however, it is clear that several of the proposed strategies have overlapping effects affecting other mechanisms

We will review various strategies for improving the homing and engraftment of HSPCs (Fig. 1), based on their classification into the following categories: i) increasing the biological effects of membrane lipid rafts, ii) modifying the expression and function of BM homing molecules, iii) modifying the metabolism of HSPCs, and iv) enhancing the availability of chemotactic factors for HSPCs. However, we are aware that this is a rather artificial classification of the strategies employed in this review and is followed for reasons of simplicity; however, several of these strategies, in fact, overlap and involve more than one category.

## Homing Strategies Based on Increasing the Biological Effects of Membrane Lipid Rafts

The cell surface membrane is a selectively permeable structure that separates the interior of the cell from the outside environment surrounding the cell. The cell membrane consists of a phospholipid bilayer, with several embedded proteins, that is held together via non-covalent interactions between the hydrophobic phospholipid tails. It has been demonstrated that, under physiological conditions, phospholipid molecules in the cell membrane are in a liquid crystalline state. However, cell membranes also contain combinations of glycosphingolipids and protein receptors organized into glycoprotein microdomains, called lipid rafts, which play an important role in several processes regulating cell biology [20, 21]. These dynamic microscopic cholesterol-enriched structures are important in assembling signaling molecules together with cell surface receptors and have been identified as playing a primary role in signaling. For example, the SDF-1-binding, G protein-coupled receptor CXCR4 must be incorporated into membrane lipid rafts for optimal association with downstream signaling proteins [22]. Thus, strategies aimed at promoting lipid raft formation could enhance the responsiveness of HSPCs to SDF-1 gradients and thereby facilitate homing of HSPCs (Fig. 1a).

### The Priming Effect of Certain Small Molecules That Increase the Responsiveness of HSCs to an SDF-1 Gradient

The SDF-1–CXCR4 axis is the most important chemotactic axis involved in the homing of HSPCs to BM [1–3]. Nevertheless, the measured SDF-1 level in the BM microenvironment after myeloblative conditioning for transplantation is relatively low (2–3 ng/ml). This level is in striking contrast to the SDF-1 doses usually employed (200–300 ng/ml) in ex vivo experimental migration assays for HSPCs, which are much too high and not physiologic. Moreover, although SDF-1 mRNA increases in the BM microenvironment after myeloablative conditioning by radio- or chemotherapy [23], this treatment induces a highly proteolytic microenvironment in BM [24]. SDF-1, as a peptide susceptible to proteolytic digestion, is a target for several proteolytic enzymes that are released from myeloid cells, and thus it becomes easily digested and inactivated [25].

Nevertheless, evidence has accumulated that this potential decrease in SDF-1 protein level in the BM microenvironment after conditioning for transplantation is somewhat mitigated by several factors, such as complement cascade (ComC) cleavage fragments (C3a and _desArg_C3a), other cationic microbial peptides released from granulocytes, such as cathelicidin (LL-37) and β2-defensin, as well as other molecules, such as hyaluronic acid, fibronectin, and soluble VCAM-1, ICAM-1, and uPAR, which may significantly enhance the responsiveness of HSPCs to a shallow SDF-1 gradient [3, 21, 22, 26–28]. This phenomenon is known in the literature as a priming effect on an SDF-1 gradient and significantly compensates for a decrease in SDF-1 level in the BM microenvironment, which occurs due to its degradation after induction of a proteolytic microenvironment. The priming effect is explained by evidence that all these small molecules promote lipid raft formation in HSPCs and thus increases HSPC responsiveness to an SDF-1 gradient via recruitment of CXCR4 [29]. Therefore, short ex vivo exposure of HSPCs to certain small molecules before infusion enhances their homing to BM. This effect has been demonstrated in vivo with C3a [30] and LL-37 [26]. Using several experimental strategies, including confocal microscopy and analysis of membrane fractions enriched in lipid rafts, it has been demonstrated that, after exposure of HSPCs to these small molecules, CXCR4 (a membrane lipid raft-associated receptor) interacts more efficiently with SDF-1 and better engages proteins in downstream signaling pathways that are involved in trafficking and adhesion of HSPCs [22, 31, 32].

The feasibility of ex vivo C3a priming of UCB cells before infusion into the patient was recently demonstrated in a clinical trial [33]. In the meantime, however, experimental evidence has accumulated that LL-37, a cathelicidin fragment, is a much more potent priming factor than C3a [26]. Moreover, since LL-37 is a physiological antibiotic peptide secreted by granulocytes and a potentially safe molecule for ex vivo graft manipulation, we propose an LL-37-based priming strategy as an interesting and simple alternative.

### Short Exposure of HSPCs to Mild Heat Treatment

Another interesting strategy to increase lipid raft formation on the surface of HSPCs, and thus facilitate homing and engraftment of these cells, has been proposed recently by another group [32]. Specifically, it was demonstrated that a short, mild ex vivo heat treatment (39.5 °C) of a UCB graft primed the human CD34^+^ cells for migration in response to an SDF-1 gradient and enhanced engraftment of these cells in immunodeficient mice.

This mild heat treatment was associated with increased expression of CXCR4 on human CD34^+^ cells and, most importantly, enhanced co-localization of CXCR4 within the lipid raft domains. CXCR4 incorporated in lipid rafts interacted better with Rac-1, a GTPase that is crucial for cell migration and adhesion [32]. Based on this intriguing observation, mild heating of umbilical cord blood-derived HSPCs before transplantation may, as proposed by the authors, become a relatively simple and inexpensive strategy to enhance homing and engraftment of transplanted cells.

## Modification of Expression and Function of BM Homing Molecules

There are several molecules on the surface of HSPCs that play a role in directing cell migration in response to BM-released chemoattractants, tethering of infused cells to the endothelium in BM sinusoids, and promoting their subsequent adhesion in the BM microenvironment. The most important receptors, besides the already mentioned CXCR4, are the type 1 receptor for S1P (S1PR_1_) and certain cell-surface mucins (e.g., P-selectin glycoprotein ligand 1 [PSGL-1]) [34]. Based on these findings, some of the strategies to improve homing have been developed based, on the one hand, on increased expression of these receptors on the cell surface and, on the other hand, on enhancing their biological pro-adhesive functions (Fig. 1b).

### Pulse Exposure of HSPCs to Prostaglandin E_2_

Prostaglandins are very short-lived molecules that belong to a large family of bioactive lipids. All nucleated cells, including those in the BM microenvironment, synthesize prostaglandins, which are involved in several pleiotropic biological effects. Prostaglandin E_2_ (PGE_2_) has been reported to be involved in regulating the biology and trafficking of HSPCs [35]. It has been shown that pulse exposure of murine or human BM cells to PGE_2_ stimulates cell proliferation, cycling, and differentiation of more primitive hematopoietic stem cells (HSCs), leading to an increase in the number of more differentiated hematopoietic progenitor cells (HPCs) [35]. These observations obtained with murine and human cells were recently validated in a zebrafish model [36]. Based on these observations, a short-pulse exposure to PGE_2_ was employed to facilitate engraftment of murine and human HSPCs. As reported, one of the effects of PGE2 is to increase expression of CXCR4 on the surface of HSPCs, which facilitates their responsiveness to SDF-1 gradients. Pre-clinical limiting-dilution studies revealed that equivalent engraftment in immunodeficient mice was achieved with 4-fold fewer PGE_2_-treated human UCB cells than with untreated control UCB cells [37]. In sum, the effect of PGE_2_ in upregulating CXCR4 receptor expression plays an important role, on the one hand, in their migratory responsiveness to an SDF-1 gradient and, on the other hand, in tethering infused HSPCs to SDF-1 expressed on the surface of endothelial cells in BM sinusoids [38]. This promising strategy has already been confirmed in a clinical study and awaits further validation [6].

### Upregulation of the CXCR4 Receptor on the Surface of HSPCs by Valporic Acid

It is obvious that any strategy that leads to upregulation of CXCR4 on the surface of HSPCs would enhance the homing effect of the SDF-1–CXCR4 axis. While potential application of CXCR4-overexpressing vectors in HSPCs is problematic because of safety issues, a relatively simple strategy has been proposed based on exposure of HSPCs to the histone deacetylase inhibitor valporic acid (VPA) [39]. It is well known that histone deacetylase inhibitors, including VPA, stimulate proliferation and self-renewal of normal HSPCs. Importantly, it has been demonstrated that exposure of murine BM mononuclear cells to VPA enhanced spleen colony formation and BM engraftment of murine HSPCs. To shed more light on this phenomenon, human UCB-derived CD34^+^ cells were exposed to VPA, and it has been observed that such exposure both upregulates CXCR4 expression on the surface of HSPCs and enhances migration of these cells up an SDF-1 gradient [39].

This relatively simple strategy to expose ex vivo HSPs to VPA has become important, because VPA has recently been successfully employed for expansion of UCB-derived primitive HSCs, which are enriched for Oct-4^+^ cells [40]. Therefore, the combined effects of VPA on upregulation of CXCR4 on the cell surface to enhance cell homing and on ex vivo expansion of HSCs could become a promising strategy for clinical application of this drug.

### Surface Fucosylation of HSPCs

As mentioned above, after infusion into the circulation, HSPCs navigate towards the BM, and the first step in the homing process is their rolling adhesions and tethering on P-selectin and E-selectin, which are expressed on the surface of endothelial cells in BM sinusoids [34]. The P- and E-selectins expressed by endothelial cells are membrane-bound lectins that interact with cell-surface glycoconjugate ligands expressed on the surface of HSPCs. In order to be biologically active, these selectin ligands have to be properly α1-3 fucosylated [34].

The most important ligand for P- and E-selectins on endothelial cells in BM sinusoids is the HSPC-expressed mucin known as P-selectin glycoprotein ligand 1 (PSGL-1). It has been reported in the case of UCB-derived HSPCs that α(1,3) fucosylation of mucins on the surface of these cells is inadequate and results in poor binding of CD34^+^CD38^–/low^ cells to E- and P-selectins in BM sinusoids [34]. To ameliorate this defect, UCB cells were exposed to guanosine diphosphate fucose in the presence of α(1,3) fucosyltransferase VI in order to fucosylate cell surface mucins, including PSGL-1. It has been reported that this ex vivo enzymatic approach enhanced engraftment of UCB cells in BM in immunodeficient NOD/SCID mice [34]. Nevertheless, this interesting strategy awaits verification in a clinical setting.

### “Painting” of HSPCs by Platelet-Derived Extracellular Microvesicles

Another approach that has been proposed to improve tethering of transplanted HSPCs to the BM endothelium is ex vivo incubation of the HSPCs in a hematopoietic graft with PB platelet-derived extracellular microvesicles (ExMVs) [41]. It has been demonstrated that ExMVs transfer not only CXCR4 but also several receptors that are crucial for adhesion of platelets to endothelium from platelets to HSPCs. Based on this effect, HSPCs isolated from murine BM or human UCB and pre-incubated with platelet-derived ExMVs engrafted much faster after transplantation into normal or immundeficient mice, respectively [41]. Furthermore, since HSPCs isolated from mobilized PB are already highly covered by platelet-derived ExMVs as a result of platelet activation in the plastic tubing during leucopheresis, this strategy is more suitable for HSPCs aspirated from BM or isolated from UCB. It has been hypothesized that, since HSPCs isolated from mobilized PB are already densely covered by ExMVs, this phenomenon may explain differences in engraftment kinetics between mPB-derived and BM-aspirated HSPCs.

## Metabolic Modification of HSPCs

The knowledge and identification of metabolic pathways that regulate self-renewal, proliferation, differentiation, and migration of HSPCs has increased significantly over the past few years. In parallel, the role of mitochondria in HSPC biology as a source of free radicals [42], the biological effects of heme oxygenase 1 [43], and the involvement of glycogen synthase kinase 3β (GSK-3β) as a regulator of the wingless (Wnt)–β catenin pathway are better understood [44, 45]. Based on this enhanced understanding, several new strategies to improve homing and engraftment have been proposed (Fig. 1c).

### Exposure of Cells in Hematopoietic Grafts to Hypoxia as a Means to Enhance Their Homing and Engraftment

It is known that HSPCs reside in specific BM stem cell niches under hypoxic conditions and rely for their metabolism on anaerobic glycolysis. As in BM, HSPCs in UCB are exposed to hypoxia. By contrast, most of the current processing strategies to prepare hematopoietic grafts from BM- or UCB-derived cells are performed under hyperoxic conditions, which leads to induction of transition pore permeability in mitochondria and results in the release of free radicals or reactive oxygen species (ROS) [42].

ROS, on the other hand, increase differentiation and cycling of HSCs, which may result in their differentiation and depletion. This effect of hyperoxia combined with release of ROS from mitochondria has been named extraphysiologic oxygen shock/stress (EPHOSS) [42]. This unwanted effect of ROS release can be prevented by collection and processing of HSPCs in low oxygen tension (3 %) in ambient air or in the presence of cyclosporin A (CyA), which protects cells from EPHOSS. In support of this concept, a beneficial effect of hypoxia and CyA was demonstrated in a recent elegant study employing an HSPC transplant model in mice [42]. This promising strategy may lead to initiation of a relatively simple, but at the same time very promising, clinical trial to enhance engraftment of UCB.

### Inhibition of Heme Oxygenase 1 (HO-1) in HSPCs

Heme oxygenase 1 (HO-1) is an inducible stress-response enzyme that not only catalyzes the degradation of heme (e.g., released from damaged erythrocytes) but also performs an important function in various physiological and pathophysiological states associated with cellular stress, such as ischemic/reperfusion injury. As already reported, HO-1 has negative effects on adhesion and migration of neutrophils in the state of acute inflammation [46], and this observation was confirmed recently for HSPCs [43]. HSPCs purified from HO-1 KO mice, and thus HO-1-deficient, show enhanced migration in response to SDF-1 and S1P gradients. This phenomenon is also supported by the highly migratory state in vivo of HSPCs from mice lacking one HO-1 allele (HO-1^+/−^). Interestingly, wild type mice transplanted with BM from HO-1^+/−^ animals showed accelerated hematopoietic recovery from myelotoxic injury compared with lethally irradiated recipients transplanted with BM cells from normal control animals [43]. Unfortunately, mice transplanted with HO-1^+/−^ HSPCs were less effective in radioprotection as well as in serial repopulation studies of myeloablated recipients.

Based on these observations, we hypothesized that transient but not permanent inhibition of HO-1, achieved by employing ex vivo exposure to small-molecule inhibitors of this enzyme, could have a beneficial effect in increasing the chemotactic responsiveness of HSPCs to SDF-1 and S1P homing gradients and that this HO-1 transient inhibition strategy could enhance homing and engraftment of transplanted HSPCs [43]. In fact, our in vitro and in vivo animal experiments demonstrated that transiently inhibiting HO-1 activity in HSPCs by small-molecule inhibitors improves HSPC homing and engraftment. We propose that this simple and inexpensive strategy could be employed in the clinical setting to improve engraftment of HSPCs, particularly in those cases in which the number of HSPCs available for transplant is limited and optimal engraftment of infused cells is desirable.

### Inhibition of GSK-3β in HSPCs to Improve Engraftment

The main purpose of hematopoietic transplantation is to transplant long-term repopulating HSCs endowed with the ability to sustain long-term hematopoiesis. Glycogen synthase kinase 3β (GSK-3β) has been identified as an important regulator of HSC function due to activation of the wingless (Wnt)–β-catenin pathway, which stimulates proliferation of HSCs [44]. Thus, Wnt–β catenin signaling plays an important role in maintaining stem cell regenerative potential under steady-state conditions. Wnt signaling enforces quiescence of HSCs in BM niches and preserves the capacity of these cells for self-renewal. This mechanism is often perturbed in cells isolated from BM or UCB and, in particular, in HSPCs after their ex vivo expansion. Therefore, a novel strategy based on ex vivo application of the GSK-3β inhibitor, 6-bromoindirubin 3′ oxime (BIO), has been proposed to delay cycling of harvested cells so that long-term repopulating HSCs will be preserved in the graft [45]. In appropriate experimental models it has been convincingly demonstrated that GSK-3β inhibition promotes engraftment of ex vivo-expanded human and murine HSPCs in normal and immunodeficient mouse models, respectively [44, 45]. However, this strategy of employing a small-molecule inhibitor of GSK-3β has not yet been tested in a clinical setting.

## Enhancing the Availability of Chemotactic Factors for HSPCs

HSPC chemotactic factors play a crucial role in BM homing and, as mentioned above, a proteolytic microenvironment induced by myeloablative conditioning affects the bioavailability of SDF-1 in BM. While we have learned a lot about induction of a proteolytic microenvironment in BM as a response to myeloablative conditioning, more work is needed to see whether, in addition to proteolytic enzymes, lipolytic enzymes are also induced and thereby affect the bioavailability of S1P and C1P. Similarly, enzymes degrading ATP could also potentially affect an ATP homing gradient. On the other hand, as will be discussed below, some of the enzymes that may affect the bioavailability of chemotactic factors are secreted by HSPCs themselves (Fig. 1d).

### Inhibition of Dipeptidylpeptidase 4 (DPP4) on the Surface of HSPCs

Dipeptidylpeptidase 4 (DPP4, also known as CD26) antigen, expressed on the surface of HSPCs, is endowed with proteolytic activity and may inactivate SDF-1 close to the cell membrane before it engages the CXCR4 receptor [47]. To ameliorate this unwanted effect, it has been proposed that blockade of DPP4 enhances the responsiveness of HSPCs to an SDF-1 gradient. In support of this hypothesis, both competitive and non-competitive hematopoietic transplants of murine and human HSPCs in normal and immunodeficient mice, respectively, revealed that a short exposure of transplanted cells to diprotinin A, which is an inhibitor of proteolytic DPP4 activity on the cell surface, enhanced homing and engraftment of these cells [47]. Thus, inhibition of DPP4 prevents SDF-1 degradation and facilitates its chemotactic interaction with the CXCR4 receptor. Most importantly, an oral inhibitor of DPP4 known as sitagliptin has been developed, and this form of DPP4 inhibitor is currently employed in clinical trials in humans transplanted with UCB [6]. This promising strategy awaits clinical verification with a larger number of patients.

### Modification of the Responsiveness of HSPCs to S1P and C1P Gradients

As mentioned above, bioactive phosphosphingolipids are potent chemoattractants for HSPCs in physiological doses and the cell-surface binding receptors for S1P are well described. These receptors are members of a family of five receptors (S1PR_1_–S1PR_5_), of which S1PR_1_ is crucial in eliciting the chemotactic responsiveness of cells to an S1P gradient [29]. By contrast, S1PR_2_ has the opposite function [29]. Therefore, one tempting strategy is to blockade S1PR_2_ by employing a specific small-molecule inhibitor, JTE-013. This strategy has already been demonstrated to improve migration of neural progenitors in response to an S1P gradient [48].

HSPCs also express lipid phosphate phosphatases (LPP1–3) on the cell surface, which possess ecto-enzymatic-degrading activity against S1P and C1P [29, 49]. Thus, LPP1–3 expressed on the surface of HSPCs could negatively affect their migration in response to gradients of C1P and S1P (dephosphorylated sphingosine and ceramide do not chemoattract HSPCs). This is a somewhat analogous phenomenon to the effect of DPP4 on SDF-1-mediated chemotaxis. Therefore, in order to increase the responsiveness of HSPCs to S1P and C1P gradients, it would be interesting to see whether ex vivo exposure of HSPCs to the LLP inhibitor XY-14 enhances engraftment of HSPCs.

Finally, since the BM level of S1P may be additionally increased by inhibiting S1P lyase (SPL) by deoxypyridoxine (DOP), a vitamin B6 antagonist, or 2-acethyl-4-tertrahydroxybutylimaidazole (THI), this inhibition should enhance the S1P level in BM and thus additionally improve the responsiveness of HSPCs to an S1P homing gradient [29]. Since inhibitors of S1P lyase (DOP and THI) are FDA-approved compounds, this relatively simple strategy for improving homing of HSPCs in patients that can be preconditioned before transplantation with an SPL inhibitor awaits experimental verification.

## Conclusions

Optimal and efficient stem cell homing will also enable achievement of the desired therapeutic effect using a lower number of stem cells. This is important, for example, in transplantation of UCB. Since the number of HSCs in UCB is limited and often insufficient to transplant to an adult recipient, strategies that enhance homing (seeding efficiency of HSCs to BM) and subsequent engraftment of UCB-derived HSCs are vital for positive clinical outcomes. In the near future we expect that some of the strategies proposed above will be widely employed or that even more novel and powerful strategies will be developed to enhance stem cell homing that target both the local niche and the stem cells employed for treatment. In addition, it is also possible to combine some of the strategies for improved homing, as discussed above. In support of this notion, inhibition of DPP4 was successfully combined with a short exposure of human or murine HSPCs to PGE_2_ to enhance their engraftment in experimental animal models [38]. Moreover new factors become identified such as for example DEK protein [50] that may ex vivo stimulate hematopoietic graft and thus enhance homing and engraftment of HSPCs. Finally, efficient homing and engraftment of stem cells employed in therapy has an immediate impact on the final outcome of the therapy not only in hematological settings but also in other potential applications of stem cells in tissue or organ injuries (e.g., to myocardium, liver, or kidney) [51].
